# How lesions at different locations along the visual pathway influence pupillary reactions to chromatic stimuli

**DOI:** 10.1007/s00417-021-05513-5

**Published:** 2021-12-13

**Authors:** Carina Kelbsch, Krunoslav Stingl, Ronja Jung, Melanie Kempf, Paul Richter, Torsten Strasser, Tobias Peters, Barbara Wilhelm, Helmut Wilhelm, Felix Tonagel

**Affiliations:** 1 University Eye Hospital, Centre for Ophthalmology, University of Tuebingen, Tuebingen, Germany; 2 Pupil Research Group at the Centre for Ophthalmology, University of Tuebingen, Tuebingen, Germany; 3 Center for Rare Eye Diseases, University of Tuebingen, Tuebingen, Germany; 4 Institute for Ophthalmic Research, Centre for Ophthalmology, University of Tuebingen, Tuebingen, Germany

**Keywords:** Chromatic pupil campimetry, CPC, Pupillometry, Chiasm, Visual pathway, Neuro-ophthalmology

## Abstract

**Purpose:**

To examine systematically how prechiasmal, chiasmal, and postchiasmal lesions along the visual pathway affect the respective pupillary responses to specific local monochromatic stimuli.

**Methods:**

Chromatic pupil campimetry (CPC) was performed in three patient groups (10 subjects with status after anterior ischemic optic neuropathy, 6 with chiasmal lesions, and 12 with optic tract or occipital lobe lesions (tumor, ischemia)) using red, low-intensity red, and blue local stimuli within the central 30° visual field. Affected areas - as determined by visual field defects revealed using conventional static perimetry - were compared with non-affected areas. Outcome parameters were the relative maximal constriction amplitude (relMCA) and the latency to constriction onset of the pupillary responses.

**Results:**

A statistically significant relMCA reduction was observed in the affected areas of postchiasmal lesions with red (*p* = 0.004) and low-intensity red stimulation (*p* = 0.001). RelMCA reduction in the affected areas seemed more pronounced for low-intensity red stimulation (46.5% mean reduction compared to non-affected areas; 36% for red stimulation), however statistically not significant. In prechiasmal lesions, a statistically significant latency prolongation could be demonstrated in the affected areas with low-intensity red stimulation (*p* = 0.015).

**Conclusion:**

Our results indicate that the choice of stimulus characteristics is relevant in detecting defects in the pupillary pathway of impairment along the visual pathway, favoring red stimuli of low intensity over blue stimuli. Such knowledge opens the door for further fundamental research in pupillary pathways and is important for future clinical application of pupillography in neuro-ophthalmologic patients. 
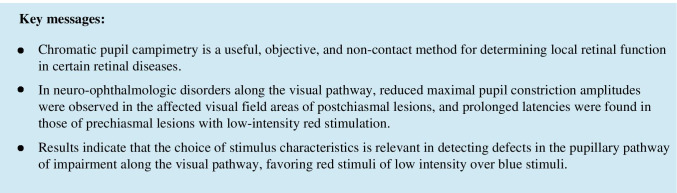

**Supplementary Information:**

The online version contains supplementary material available at 10.1007/s00417-021-05513-5.

## Introduction

Color pupillography is a useful, objective, and non-contact method for determining retinal function in certain retinal diseases. Full-field pupillography allows for the evaluation of the summed response of the entire neuroretinal function, e.g., in retinitis pigmentosa [1–4]. However, spatial pupillographic strategies, such as chromatic pupil campimetry (CPC) [5, 6], are of increasing importance, as they objectively provide information of local functionality for natural history observations as well as follow-up after therapeutic intervention in patients with specific retinal diseases. Defects on retinal level, e.g., rod-cone dystrophies or age-related macular degeneration, are precisely detectable by CPC [6–9].

This raises the question how do defects in the subsequent network of the visual pathway affect pupillary responses? Several studies with different devices and strategies have proven that focal pupillometry can successfully map certain visual field defects [5, 10–16]. However, in some cases, visual field and pupillographic results are perfectly matching, and in others, they are not. Currently, the underlying mechanism of this discrepancy is not fully understood. Bremner et al. proposed different visual and pupillary pathways with a “pupil sparing” in hereditary optic neuropathies such as Leber hereditary optic neuropathy (LHON) and autosomal dominant optic atrophy (ADOA) (i.e., pupil reaction is relatively better preserved than the visual field) and a “visual sparing” in demyelinating optic neuritis (i.e., visual field is relatively less affected than the pupil reaction) [17].

The pupillary pathway starts in photosensitive retinal cells - rods, cones, and melanopsin-containing intrinsically photosensitive retinal ganglion cells (ipRGCs) - and passes through the axons of the retinal ganglion cells forming the optic nerve, the chiasm, and optic tract. Before reaching the lateral geniculate nucleus (LGN), pupillomotor axons are separating from visual axons and synapsing in the olivary pretectal nucleus (OPN) in the dorsal midbrain. Based on primate studies, pupillary fibers are supposed to arise predominantly from ipRGCs via the extrinsic pathway from rod/cone input and the intrinsic melanopsin pathway [18]. From the OPN, both Edinger-Westphal nuclei are targeted, the parasympathetic subnuclei of the oculomotor nerve, whose efferent fibers conduct the light signal to the pupil sphincter and elicit the pupil light reaction (PLR) [19, 20]. Besides this classical subcortical pupillary pathway, retrogeniculate and cortical impact on the PLR is evident [11, 21–24], but still not entirely understood. Besides the influences of these pathways on the formation of the PLR, it was recently shown that acquired occipital lobe lesions leading to homonymous hemianopia can be followed by retrograde retinal ganglion cell damage and atrophy in the retinal half side that corresponds to the blind visual field [25, 26]. The effect of such retrograde retinal ganglion cell damage on the PLR has to our knowledge not been systematically explored. Furthermore, there are indications to the existence of an additional, melanopsin-mediated pupillary pathway circumventing the dorsal midbrain [27].

The aim of this study was to exploratively and systematically measure pupillary responses to local monochromatic red and blue stimuli within the central 30° visual field with CPC in patients suffering from impairment at specific locations along the visual pathway. We examined how prechiasmal lesions of the optic nerve and chiasmal lesions and postchiasmal lesions of the optic tract and occipital lobe affect the respective pupillary responses.

## Methods

### Participants

Twenty-eight adult volunteers were recruited via the Neuro-Ophthalmology Department of the University Eye Hospital Tübingen by experienced neuro-ophthalmologists. During the neuro-ophthalmologic clinical routine examination, all consecutive suitable candidates meeting the subsequent inclusion criteria were asked for voluntary participation in the pupillographic study and categorized into three groups with specific defects of the visual pathway:

#### Group 1 prechiasmal lesions

Group 1 prechiasmal lesions include 10 subjects (9 male; 61 ± 10 years; mean ± SD) with confirmed status after anterior ischemic optic neuropathy (AION) showing partial optic nerve atrophy in optical coherence tomography (Spectralis-OCT, Heidelberg Engineering GmbH, Heidelberg, Germany).

To ensure a homogenous patient cohort and exclude possible disturbing influences of different entities of prechiasmal lesions, only the well-diagnosed confirmed status after AION was considered eligible for participation. The diagnoses of AION were based on the characteristic anamnesis of a sudden painless onset of visual disturbance with typical clinical findings of sectorally emphasized papilledema followed by corresponding partial optic nerve atrophy as evaluated by experienced neuro-ophthalmologists.

#### Group 2 chiasmal lesions

Group 2 chiasmal lesions include 6 subjects (3 male; 52 ± 17 years) with confirmed chiasmal lesions (suprasellar tumor in cerebral imaging).

#### Group 3 postchiasmal lesions

Group 3 postchiasmal lesions include 12 subjects (10 male; 58 ± 14 years) with homonymous hemi- or quadrantanopia due to confirmed optic tract lesions (tumor, ischemia, trauma) or occipital lobe lesions (tumor, ischemia) in cerebral imaging.

The exclusion criteria were as follows:

- Abnormal pupil light reaction due to iris damage or disturbance in nerve supply
- Diseases of the eye and/or general diseases and/or medication influencing the pupils’ mobility or severe visual impairment due to severe cataract or corneal scars
- Additional eye diseases influencing the retinal function (e.g., glaucoma, retinal detachment, high myopia) or which interfere with the measurement (e.g., nystagmus)

Detailed demographic and neuro-ophthalmologic clinical data including visual field defects, optic atrophy in OCT (standard circle scan), and diagnoses are presented in Online Supplement Table 1.

Post-study, in postchiasmal lesions, additional OCT evaluation of the ganglion cell layer was performed; respective data were available in 6 out of 12 patients of group 3.

### Chromatic pupil campimetry (CPC)

Pupillary responses were elicited by using chromatic pupil campimetry (CPC) in a slightly modified version than previously described in detail [5, 6]. This device is a custom-built pupillograph using an OLED monitor for stimulus presentation within the central 30° visual field (Samsung/LG OLED 55C7V; Samsung, Seoul, South Korea; full HD 3840 × 2160 pixels) at a fixed distance of 40 cm to the subject’s eye. An infrared camera with a temporal resolution of 10 ms (DMK23UV024, The Imaging Source GmbH; with a 50-mm TV lens 1:1.8) allows for a precise continuous recording of the pupil diameter. A dim central fixation mark at the monitor served as a fixation target (0.01 cd/m^2^). Furthermore, gaze-tracking was used to correct for subtle eye movements, therefore allowing for a retinotopic stimulus presentation. Pupil size was calculated in real time by in-house software as described before [5]. The examination was performed in a completely dark and quiet room; one eye was patched and the other eye was stimulated and the direct pupil responses were recorded.

### Stimulus characteristics

Stimuli were presented on the OLED monitor at 25 different locations of the visual field at different eccentricities from the center (1 central location), at 6° (4 locations), at 12° (8 locations), at 20° (4 locations), and at 30° eccentricity (8 locations). The stimulus grid is shown in Online Supplement Fig. 1. Stimuli were repeated at least twice per location and the average pupillary response was calculated per location. In case of blinking artifacts during stimulus presentation, or if at least 90% of the initial baseline diameter was not reached before stimulus presentation, stimulus repetition was automatically triggered by the software.

Monochromatic cell-specific stimuli were applied according to the following scheme:

1. Red stimulation (targeting (M-/) L-cones):
  Stimulus radius 3°, stimulus intensity 60 cd/m^2^, stimulus wavelength 620 ± 30 nm FWHM (full width at half maximum), irradiance 8.2 × 10^−4^ W/m^2^, peak energy 14.765 × 10^−3^ mW/m^2^, on dim blue background (0.01 cd/m^2^; wavelength 460 nm ± 30 nm FWHM, 2.1 × 10^−8^ W/m^2^)
  This stimulus protocol has been validated showing good test-retest reliability before (mean intraclass correlation ICC: 0.75 ± 0.11) [6].
2. Low red stimulation (targeting (M-/) L-cones):
  Same characteristics as for red stimulation but with a decreased stimulus intensity of 20 cd/m^2^ (irradiance 2.96 × 10^−4^ W/m^2^, peak energy 5.692 × 10^−3^ mW/m^2^)
3. Blue stimulation (targeting S-cones, with input from melanopsin and rods): Stimulus radius 3°, stimulus intensity 20 cd/m^2^, stimulus wavelength 460 ± 30 nm FWHM, irradiance 11.11 × 10^−4^ W/m^2^, peak energy 31.2 × 10^−3^ mW/m^2^, on dim blue background (0.01 cd/m^2^; wavelength 460 nm ± 30 nm FWHM, 2.1 × 10^−8^ W/m^2^)

Further information on the energy and spectrum with CIE color coordinates of the applied stimuli are provided in Online Supplement Fig. 2.

For all stimuli, the baseline pupil diameter was recorded for 500 ms before stimulus presentation, stimulus duration was 1 s, and the interstimulus interval between the end of one stimulus and the beginning of the next stimulus was 4.5 s, in accordance with current standards in pupillography [28]. The whole examination time lasted around 15 min per eye preceded by an additional 5-min adaptation time to the background.

### Data analysis and statistics

Artifact elimination was done by linear interpolation with an in-house created software (The MathWorks, Inc. Matlab, for more details, Stingl et al. 2018 [5]).

Pupillary response parameters for the statistical analysis were the relative maximal constriction amplitude (relMCA; normalized to baseline diameter in percent) and the latency to constriction onset (latency; in ms). Latency was determined as the time from the beginning of stimulus presentation until the time of intersection of a linear fitting curve through the descending part of the pupillogram with the baseline. For the determination of the fitting curve, 20 points around the turning point of the pupillogram were chosen using an in-house created algorithm (The MathWorks, Inc. Matlab).

For red and low red stimulation in postchiasmal lesions, additional calculation of the mean relMCA reduction of affected areas in percent from non-affected areas was reported and compared: [relMCA_(non-affected)_ – relMCA_(affected)_] / relMCA_(non-affected)_ × 100.

**Fig. 1:**
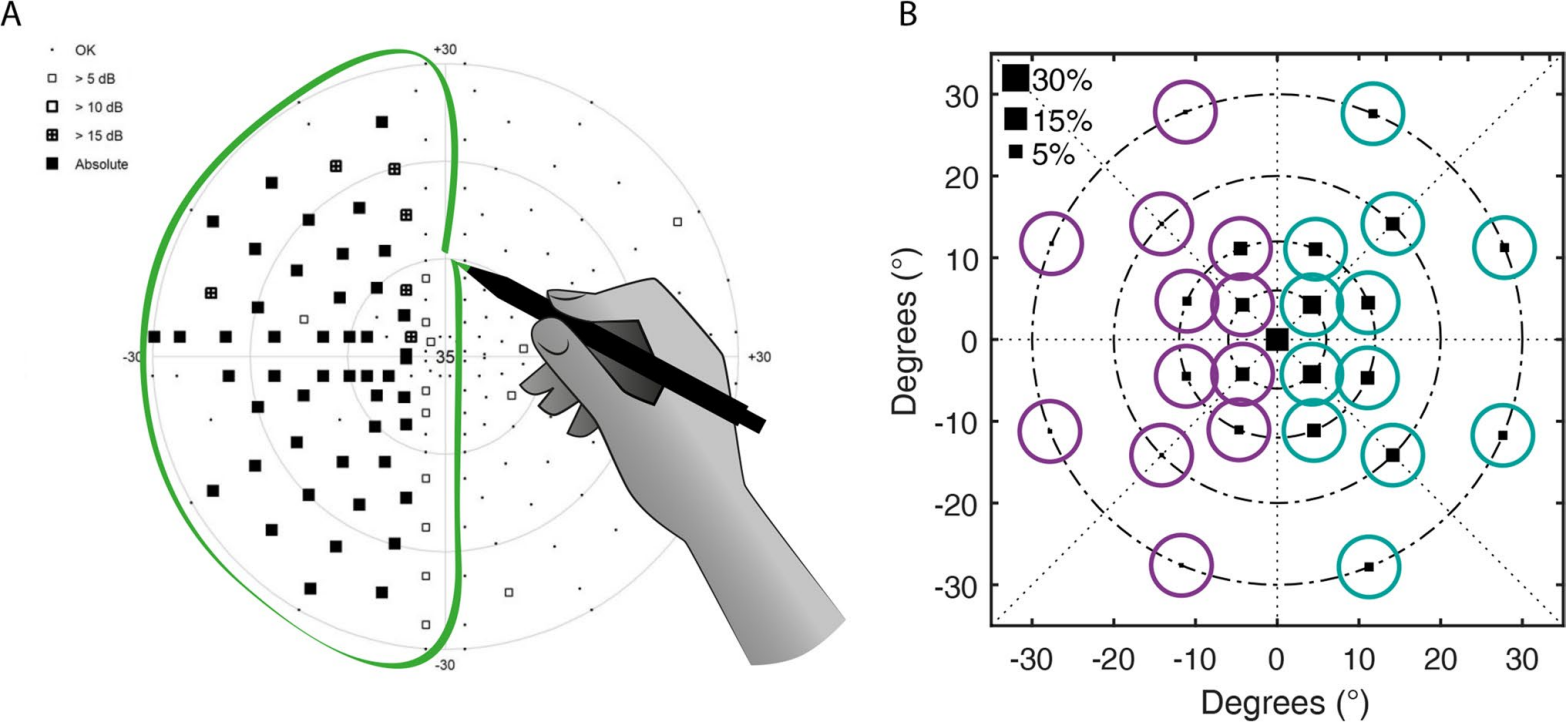
Process of categorizing pupil data from CPC according to the visual field defect in conventional perimetry. **A** Marking of the visual field defect by two experienced neuro-ophthalmologists. Black squares on the left hemifield correspond to absolute visual field defects; small black dots on the right hemifield mean normal visual field. **B** Marking of the corresponding CPC stimulus locations within the affected visual field defect to group I (violet) and those within the intact, non-affected visual field to group II (blue). The size of the black squares in the CPC data (B) represents the amount of pupil constriction per stimulation location; the bigger the square, the stronger the pupil constriction (for a reference, square sizes for 5%, 15%, and 30% relative constriction amplitudes to baseline pupil diameter are shown). However, the amount of pupil responses per location had no influence on the group categorization which was purely based on the perimetric visual field defects

Two experienced neuro-ophthalmologists (CK and FT) evaluated independently the results of conventional static perimetry (Octopus 900, Haag-Streit International, Wedel, Germany, slightly suprathreshold test strategy) and sectioned the visual field in “affected” versus healthy “non-affected” areas for each subject. No discrepancies occurred. Based on this visual field categorization, CPC locations were accordingly assigned either to affected or non-affected areas of the visual field (see Fig. 1). Afterwards, mean pupillary responses from “affected” and “non-affected” areas were compared and statistically analyzed. Responses from central stimulation (0°) were not included into the analysis because this location lies exactly on the midline of defects caused by retrochiasmal and chiasmal lesions and, furthermore, to not induce a bias to one categorization, as foveal stimulation usually has the strongest maximal constriction amplitude. In the analysis of “non-affected area”, pupillary responses from all healthy non-affected areas from all three groups were evaluated and averaged, as no homogeneous distribution nor equal number and eccentricities of stimulus locations of affected and non-affected areas was present in all individual subjects, particularly in prechiasmal lesions due to the nature of the disease.

In general, results are presented in mean ± standard deviation (SD). Two-tailed *t* tests were performed to test for differences between affected and non-affected visual field areas. To correct for multiple comparisons, the alpha level was set to 0.017 (= 0.05/3: Bonferroni correction for multiple testing for red, low red, and blue stimulation).

## Results

**Fig. 2:**
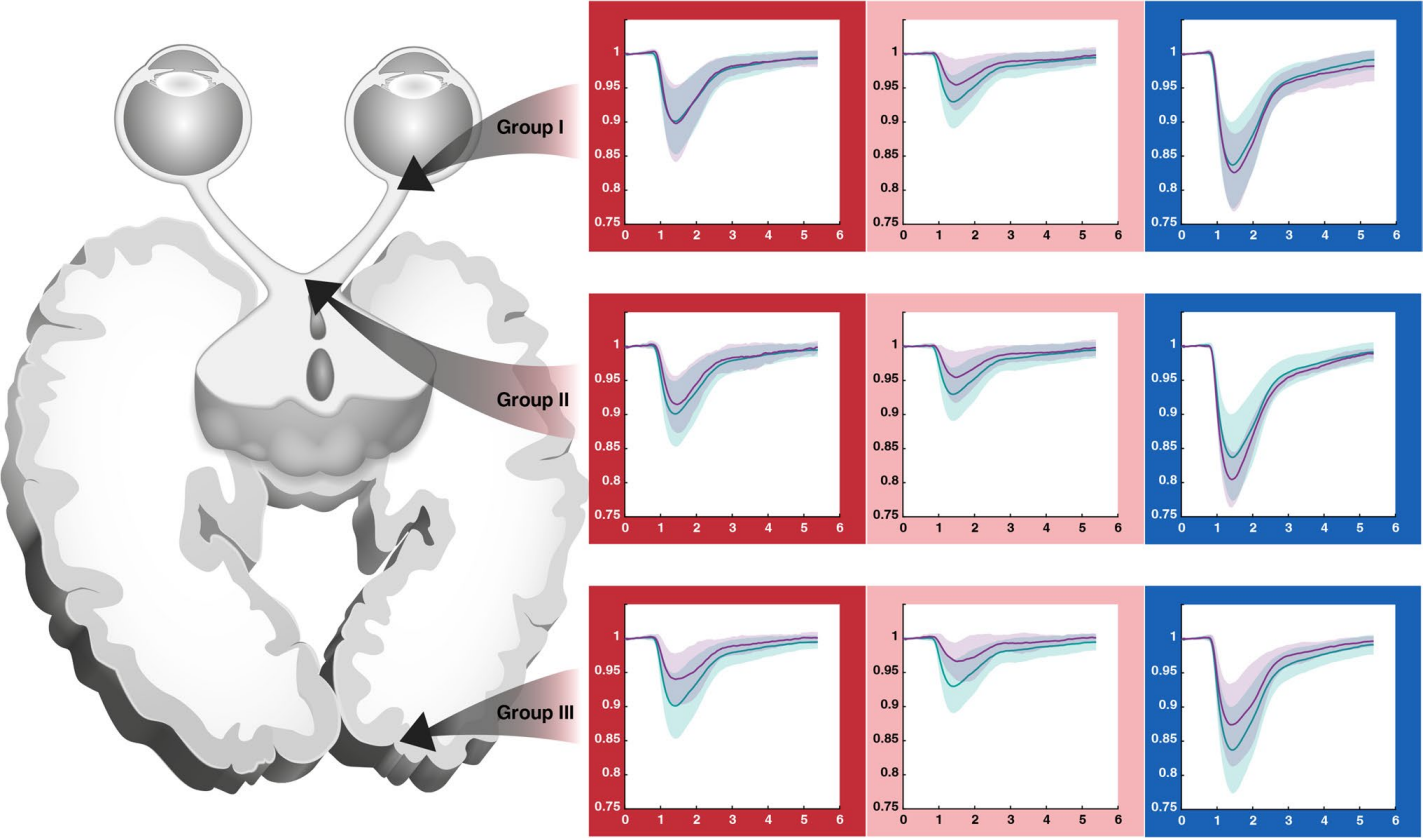
Mean pupillary responses normalized to baseline from all affected areas - as categorized by visual field defects - from all participants of groups 1–3 (violet lines) versus mean pupillary responses from all non-affected areas from all participants (blue lines) for red, low red, and blue stimulation (from left to right). The respective standard deviations are depicted as background shading in the respective colors. X-axis time in seconds, y-axis relative pupil diameter. Group 1, prechiasmal lesions: anterior ischemic optic neuropathy; group 2, chiasmal lesions; group 3, postchiasmal lesions of the optic tract and occipital lobe

Mean relMCAs and latencies from all affected areas - as categorized by visual field defects - were calculated per group (prechiasmal, chiasmal, postchiasmal) and per stimulation characteristic and compared with the respective mean pupillary responses from all non-affected areas from all participants. No statistically significant relMCA differences in the non-affected areas were found between the groups for all stimulation characteristics. Data of the analysis of the specific, monochromatic stimuli are presented in Table 1. Figure 2 shows the mean pupillary responses for affected versus non-affected areas for different stimulation characteristics separately for prechiasmal, chiasmal, and postchiasmal lesions along the visual pathway (Fig. 2).

**Table 1:** Statistical analysis for maximal constriction amplitude and latency to constriction onset for different stimulation characteristics in prechiasmal, chiasmal, and postchiasmal lesions. The mean of affected visual field areas per group is compared with the mean of non-affected areas of all subjects

	Red relMCA (%) (Mean ± SD)	Red Latency (ms) (Mean ± SD)	Low red relMCA (%) (Mean ± SD)	Low red Latency (ms) (Mean ± SD)	Blue relMCA (%) (Mean ± SD)	Blue Latency (ms) (Mean ± SD)
Pre	10.32 ± 5.93 *p* = 0.871	340.45 ± 73.79 *p* = 0.028	6.93 ± 3.80 *p* = 0.564	349.46 ± 39.97 *p* = 0.015*	17.68 ± 5.91 *p* = 0.790	292.75 ± 31.30 *p* = 0.065
Chiasmal	8.80 ± 4.72 *p* = 0.370	326.31 ± 52.75 *p* = 0.063	5.54 ± 4.84 *p* = 0.185	349.58 ± 79.15 *p* = 0.065	19.68 ± 4.40 *p* = 0.328	257.48 ± 15.16 *p* = 0.517
Post	6.78 ± 4.58 *p* = 0.004*	310.09 ± 88.18 *p* = 0.072	4.10 ± 3.56 *p* = 0.001*	345.23 ± 120.35 *p* = 0.054	13.16 ± 6.59 *p* = 0.019	279.46 ± 38.16 *p* = 0.309
Non-affected	10.60 ± 4.86	271.67 ± 73.60	7.67 ± 4.05	298.96 ± 61.99	17.15 ± 5.98	268.55 ± 40.79

*t* test, *relMCA* relative maximal constriction amplitude; significant *p* values are marked in bold and with an asterisk*

The alpha level was set to 0.017 (= 0.05/3: Bonferroni correction for multiple testing for red, low red, and blue stimulation)

### Reduction of the constriction amplitude is observed in postchiasmal lesions with red and low-intensity red stimulation

A statistically significant relMCA reduction was exclusively observed in the affected areas of postchiasmal lesions with red (relMCA affected 6.78 ± 4.58% versus non-affected 10.60 ± 4.86%; *p* = 0.004) and low red stimulation (4.10 ± 3.56% versus 7.67 ± 4.05%; *p* = 0.001). RelMCA reduction in the affected areas seemed more pronounced for low red stimulation (46.5% mean reduction compared to non-affected areas; for red stimulation mean relMCA reduction was 36%), but this effect was statistically not significant (*p* = 0.2).

For blue stimulation, a similar trend of reduced relMCAs was observed for postchiasmal lesions but missed statistical significance (*p* = 0.019). Prechiasmal and chiasmal lesions did not show significant differences between relMCAs of affected and non-affected areas.

### Prolongation of the latency to constriction onset is observed in prechiasmal lesions with low-intensity red stimulation

A significant latency prolongation could be demonstrated in the affected areas of prechiasmal lesions with low red stimulation (349.46 ± 39.97 ms versus 298.96 ± 61.99 ms for non-affected areas; *p* = 0.015).

### Postchiasmal lesions: comparison of pupillary constriction amplitudes and retrograde ganglion cell loss in OCT

**Fig. 3:**
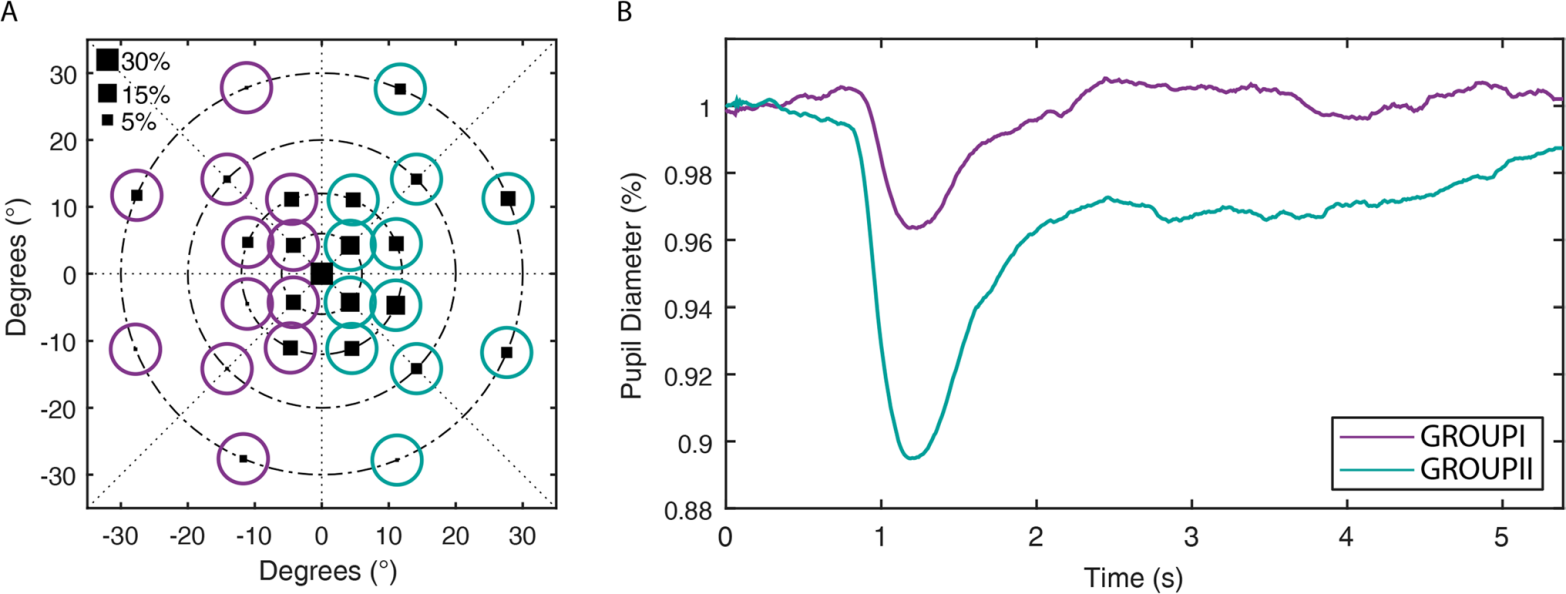
Results from chromatic pupil campimetry with low red stimulation of the right eye from patient post-03 with left homonymous hemianopia due to occipital glioblastoma demonstrating a clear reduction in the relative maximal constriction amplitudes (relMCA, normalized to baseline pupil diameter) in the affected left visual field. **A** The visually affected left hemifield is marked with violet circles, and the non-affected areas are marked with blue circles and compared with each other. The size of the black squares represents the amount of pupil constriction per stimulation location; squares increase with increasing strength of the relMCA (for a reference, square sizes for 5%, 15%, and 30% relative constriction amplitudes to baseline pupil diameter are shown). **B** The average pupil response of all stimulus locations within the visually affected left hemifield (violet) is compared to the non-affected areas (blue, right hemifield) revealing strong differences between the hemifields - a strong relMCA reduction in the affected visual field

**Fig. 4:**
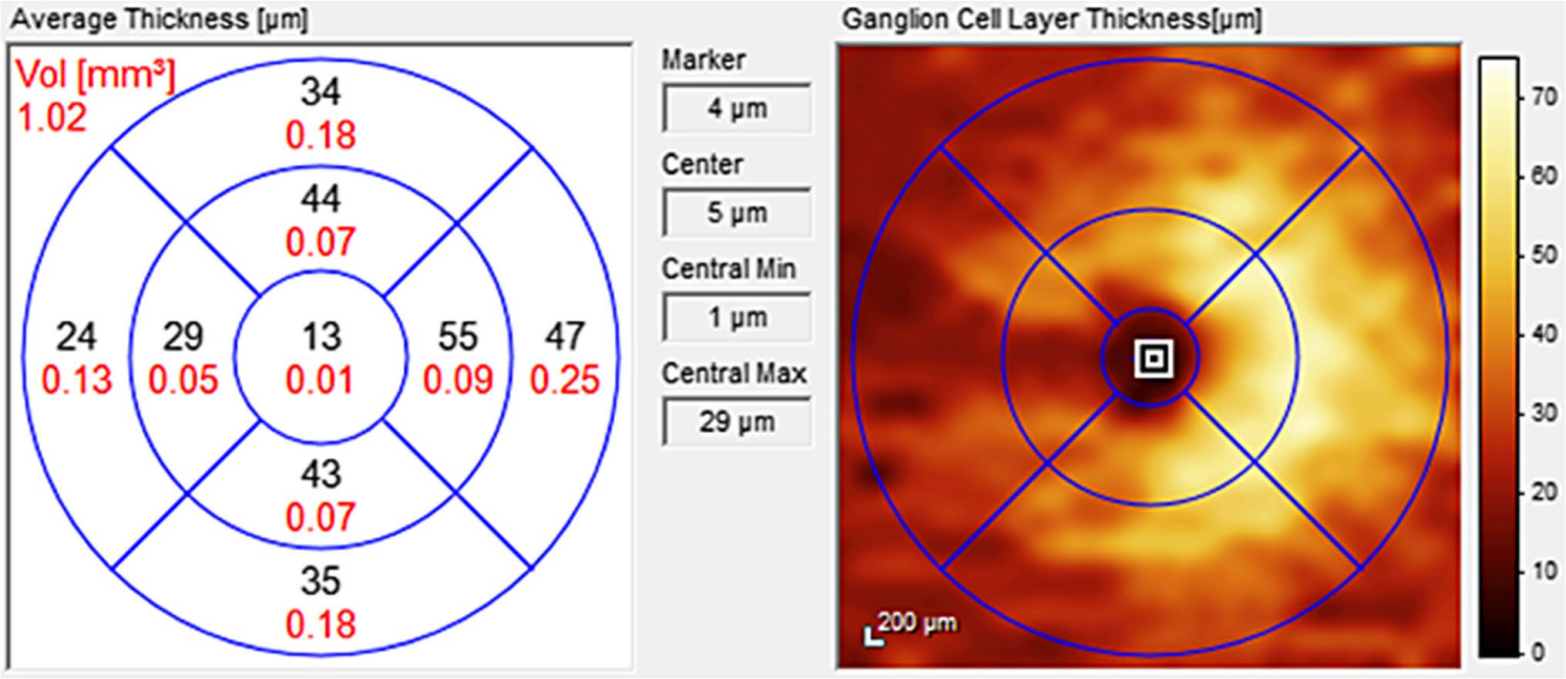
Corresponding ganglion cell layer (GCL) thickness of the macular region measured by spectral-domain-OCT of the right eye of patient post-03 with left hemianopia. A reduction of the GCL in the affected temporal hemifield is observed (temporal sector GCL 24–29 µm versus nasal sector 47–55 µm)

The individual results of the right eye for low red stimulation from patient post-03 with left homonymous hemianopia due to occipital glioblastoma are exemplarily shown in Fig. 3, demonstrating a clear reduction in relMCAs in the affected left visual field. However, such clear mapping between visual and pupillomotor defects could not be proven for all subjects - some even showed nearly no congruence. In the case of patient post-03, a corresponding reduction of the ganglion cell layer (GCL) thickness in the affected retinal hemifield could be observed in OCT, presumably through retrograde transsynaptic degeneration over time (Fig. 4). Overall, data on GCL thickness was available for 6 out of 12 patients with postchiasmal damage, revealing a clear GCL reduction in the affected hemifield in the 3 cases of them with lesions > 1 year and a slight reduction in the 2 cases with lesions being 6–8 months old. The fresh lesion with subacute ischemia approximately around 2 weeks old (post-01) was associated with a comparable GCL thickness in the affected and non-affected hemifield. Likewise, pupil responses were preserved (Table 2).

**Table 2:** Comparison of ganglion cell layer thickness and pupil constriction amplitudes in affected and non-affected hemifields of patients with postchiasmal lesions

	GCL thickness affected area	GCL thickness non-affected area	relMCA affected area	relMCA non-affected area	Approximate time from lesion onset
Post-01	R 47 µm L 47 µm	R 45 µm L 45 µm	R 12% L 12%	R 13% L 13%	2 weeks
Post-03	R 29 µm L 31 µm	R 55 µm L 48 µm	R 4% L 2%	R 11% L 6%	2 years
Post-04	R 42 µm L 43 µm	R 52 µm L 46 µm	R 8% L 9%	R 14% L 12%	8 months
Post-06	R 15 µm L 18 µm	R 58 µm L 51 µm	R 1% L 4%	R 6% L 6%	14 years
Post-10	R 20 µm L 22 µm	R 43 µm L 46 µm	n.a L 2%	n.a L 6%	Presumably months-years
Post-12	R 39 µm L 36 µm	R 42 µm L 43 µm	R 2% L 4%	R 4% L 8%	6 months

*GCL* ganglion cell layer measured by Spectralis-OCT (optical coherence tomography) at a 3 mm circle ETDRS nasal and temporal sectors, respectively, according to visually affected and non-affected visual hemifields; *relMCA* relative maximal pupil constriction amplitude

## Discussion

Whereas retinal defects are quite precisely detectable by pupillography, inconsistent results are reported in the literature with regard to defects in the subsequent visual pathway via the optic nerve, optic tract, and visual cortex. Pupil assessment in optic nerve disorders showed a “pupil sparing” in LHON and ADOA and a “visual sparing” in demyelinating optic neuritis [17], a phenomenon which is also detectable in the clinical examination of these patients. Consequently, a specific arrangement and selective deterioration of visual and pupillary fibers in the optic nerve and further pathways could be assumed, e.g., by involving different subclasses of retinal ganglion cells or further projections. Such relative preservation of ipRGCs could be histopathologically demonstrated in human retinae of mitochondrial damage in hereditary optic neuropathies (LHON, ADOA) [29].

To explore the application of pupillography in neuro-ophthalmologic patients, this study addressed the systematic, explorative measurement of pupillary responses to local monochromatic red, low-intensity red, and blue stimuli with chromatic pupil campimetry (CPC) in patients with prechiasmal lesions after anterior ischemic optic neuropathy, chiasmal lesions, or postchiasmal lesions. Our results show the relevance of carefully chosen stimulus characteristics regarding wavelength and intensity in the detection of such lesions along the pupillary/visual pathway. Stimulation with red light of low intensity (20 cd/m^2^) was superior to blue stimuli.

### RelMCA reduction in postchiasmal lesions for low-intensity red and red stimulation and the possible impact of retinogeniculostriate pathways and retrograde degeneration

In postchiasmal lesions, the maximal constriction amplitude was predominantly affected with a significant relMCA reduction in the affected areas in comparison with the non-affected areas of the visual field of all participants (*p* = 0.001 for low red stimulation). With red stimulation, the difference was likewise significant, and with blue stimulation, no significant difference between affected and non-affected areas could be demonstrated. These data underline the suitability of pupillography but also the importance of a careful selection of stimulus characteristics. One could speculate whether red stimulation, which is predominantly L-cone driven, might receive more modifying cortical influence on the PLR than blue (S-cone (and melanopsin-driven)) stimulation. Similar results of more affected pupillary responses to red compared with blue stimulation were previously found for full-field stimulation in hemianopic patients in comparison to a normal cohort [30]. Several subclasses of retinal ganglion cells (RGC) have been identified and the retinogeniculostriate pathways are distinct for M/L-cones and S-cones. M/L-cones are supposed to predominantly connect via midget RGCs to the parvocellular layers of the dorsal lateral geniculate nucleus (dLGN) and to reach layer 4 of the primary visual cortex V1. S-cones, however, synapse with small bistratified RGCs to the koniocellular layers of dLGN and layers 1 and 3 of V1 [31–33]. In addition, all cone types provide synaptic input to intrinsically photosensitive RGCs that are crucial for eliciting the PLR through their projection to the olivary pretectal nucleus (OPN) in the dorsal midbrain [18, 34, 35]. Moreover, V1 is supposed to project to OPN [35].

As blue and red stimulation follows different retinogeniculostriate pathways, cortical occipital lesions may have a stimulus-dependent impact on the PLR, respectively. There is also the observation of transsynaptic retrograde degeneration of RGCs after acquired occipital lobe lesions which is quantitatively detectable by optical coherence tomography. Such a relationship has been reported in literature [25, 26] and was also present in several patients of our cohort with longer lasting lesions, e.g., patient post-03. As GCL thinning after acquired occipital lobe lesions is time-dependent, the initial change in PLR in some cases might be driven by cortical circuits, and over time, the retrograde change in RGCs additionally could be a source of PLR impairment in the affected hemifield. However, currently, it remains unclear which role such retrograde RGC loss plays with regard to the PLR. Predominantly conventional RGC loss would presumably much less affect the PLR than a predominant loss of ipRGCs, which are supposed to carry most signals for the PLR to the OPN. Different ophthalmologic pathologies were previously shown to affect conventional RGCs and ipRGCs to different extents. For example, damage in glaucomatous optic neuropathies seems to affect particularly the melanopsin-mediated function of ipRGCs already in earlier stages of the disease [36–38]. On the other hand, mitochondrial dysfunction in hereditary optic neuropathies reveals a histopathologically and clinically clear relative resistance of ipRGCs compared to conventional RGCs [29, 39] and thus may explain the “pupil sparing” in these patients despite severe vision impairment.

Furthermore, ipRGCs likely project to the superior colliculus [34] being supposed to interact with higher cortical visual areas circumventing V1 (extrastriate pathway) [24, 40] that in turn may interact with the Edinger-Westphal nuclei in the control of the PLR, particularly in response to color, gratings, and movement [24].

### Stimulus intensity in the detection of postchiasmal lesions

Even though stimulation with low red stimuli revealed a significant relMCA reduction in several patients (see example patient in Fig. 3 and Table 2) and in the group comparison of patients suffering from postchiasmal lesions, some individuals still showed nearly unremarkable pupillary responses or a different pattern than the visual affection. Thus, normal pupillary responses in both hemifields do not automatically exclude morphological damage to the postchiasmal visual pathway and a thorough clinical work-up including cerebral imaging is necessary for neuro-ophthalmologic patient care. We consider that besides the wavelength, also stimulus intensity matters and that the low red stimulus intensity still may have been too strong for some individuals. It was previously reported that in patients with acquired damage of the primary visual cortex, pupillary responses were only absent or reduced in amplitude in the blind hemifield when stimulated with small stimuli of low luminance contrast [24]. The PLR to short stimuli of low luminance contrast seems to require both a normal afferent pathway from the retina to the OPN in the dorsal midbrain and a normal signal processing along the geniculostriate pathway. In case one of these two signal pathways is disturbed, pupillary responses will be affected. However, when stimulating with large light flux increments, the subcortical pathway to the OPN seems to become sufficient for eliciting a (near-) normal PLR as pupillary responses were reported to be similar in the blind and sighted hemifields of occipital damage [24].

### RelMCA in chiasmal and prechiasmal lesions was not significantly reduced

Visual field defects caused by chiasmal and prechiasmal lesions could not be convincingly demonstrated by relMCAs in CPC. Perhaps in cases of in concordance between pupillary responses and conventional perimetry, a “pupil sparing” as seen in hereditary optic neuropathies was the underlying effect. Kawasaki et al. presented data of hereditary optic neuropathies with similar pupillary responses between patients with mild-to-moderate visual dysfunction and controls but an association between the degree of visual field loss and the half-max intensity of the cone response to red light stimulation, indicating disturbed PLR in more advanced stages of disease [39]. Consequently, pupillary constriction amplitude does not seem to be the ideal parameter for certain optic neuropathies.

### Latency prolongation in prechiasmal lesions for low-intensity red stimulation

Indeed, in prechiasmal lesions, our results indicate a more relevant role of impaired time dynamics of the PLR. A significant latency prolongation could be demonstrated in the affected areas of prechiasmal lesions with low red stimulation (see Table 1; *p* = 0.015) - again favoring red stimuli of low intensity. Also, in chiasmal and postchiasmal lesions, a clear tendency to longer latencies in the affected areas was observed, although statistical significance was not reached (*p* = 0.065 and 0.054 respectively).

Supporting our results of a decisive role of the analysis of temporal dynamics of the PLR, we have recently observed latency as an important potential outcome parameter for retinal function in active exudative age-related macular degeneration demonstrating a similar prolonged latency in the affected areas [8]. Moreover, latency was proposed to be a suitable outcome measure of cone function after full-field stimulation in *CEP290-* or *NPHP5-*associated Leber congenital amaurosis [41]*.*

## Limitations

The limitations of our study are the relatively small number of patients especially in the group of chiasmal lesions. Therefore, no statistical comparisons have been performed to analyze for differences between the groups. Instead, the focus of the study was to compare pupillary responses from affected versus healthy non-affected areas for different stimulation characteristics. Our study was conducted as an explorative study in patients of disturbances at different locations of the visual pathway; the results raise ideas for future investigations aiming to ultimately establish an objective clinical test for neuro-ophthalmic patients, which cannot yet be derived from the current data. Retrospectively, we would have included a third red stimulus of even lower intensity and additional ganglion cell layer thickness measurements for all participants with postchiasmal lesions. Furthermore, a prospective study of patients with acute occipital stroke is desirable to evaluate pupillary responses over a time span from fresh lesion up to 1–2 years as a long-lasting disease may differently affect the pupillary response compared to a recently diagnosed disease which is a limitation of the current study.

In conclusion, our data contribute to basic research and understanding of pupil regulation as well as to clinical applicability of pupillography. For pupillography in patients with prechiasmal anterior ischemic optic neuropathies, chiasmal lesions, or hemianopia due to postchiasmal lesions, red stimuli of low intensity seem favorable. Characteristic changes are a reduction of amplitude in postchiasmal lesions and a prolonged latency to constriction onset in prechiasmal lesions. Normal pupillary responses do not principally exclude morphological damage to the visual pathway, because the PLR receives input from a more complex system, including the impact of different RGC classes, retrograde degeneration, and influences from the retinogeniculostriate and extrastriate pathways which may be impaired in different ways in lesions at different locations along the visual and pupillary system for different stimulation characteristics. Such knowledge will open the door for further fundamental research on pupillary pathways and for future clinical application of pupillography in specific neuro-ophthalmologic patients.

## Supplementary Information

Below is the link to the electronic supplementary material.

Supplementary file1 (PDF 261 KB)

Supplementary file2 (PDF 61 KB)
