# Supplementary material for: How lesions at different locations along the visual pathway influence pupillary reactions to chromatic stimuli
Source: Graefes Arch Clin Exp Ophthalmol. 2021 Dec 13;260(5):1675–85. doi: 10.1007/s00417-021-05513-5 (PMC9007757; doi:10.1007/s00417-021-05513-5)
Supplement: Supplementary file 1 — Supplementary file1 (PDF 261 KB) [file 417_2021_5513_MOESM1_ESM.pdf]

Supplement Figures

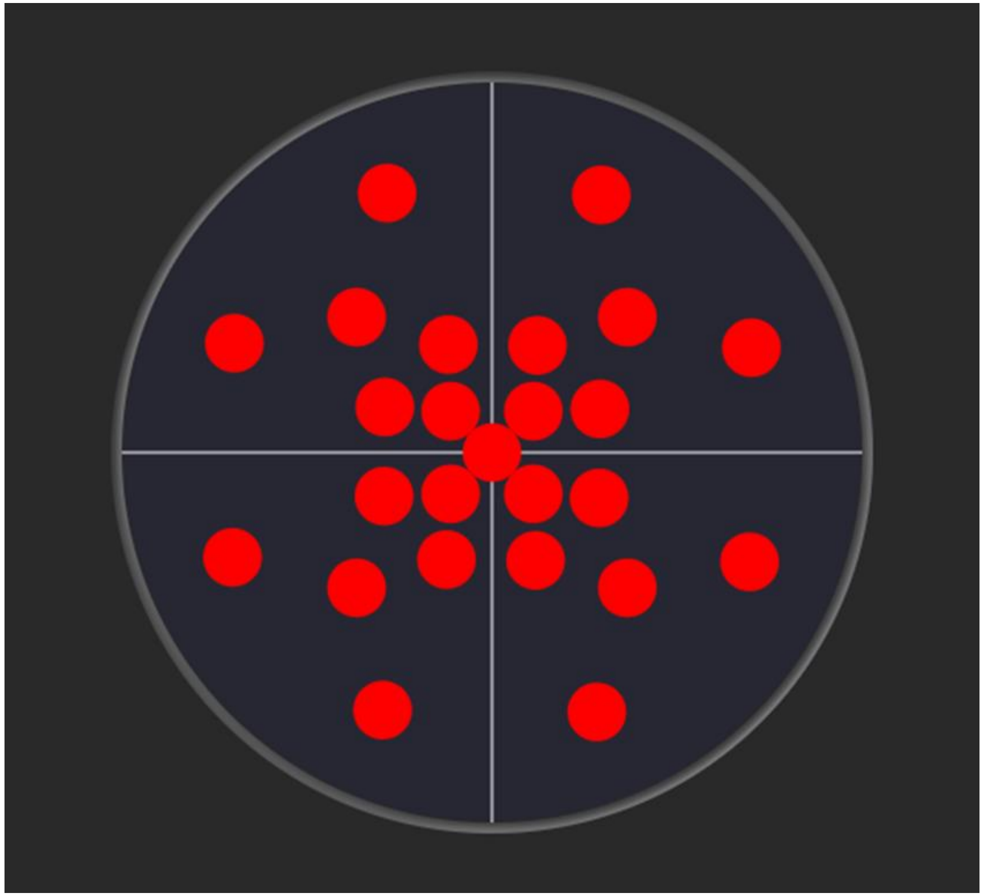

Supplement Figure 1 Stimulus grid of the 25 stimulus locations within the central 30° visual field at different eccentricities from the center (1 central location), at 6° (4 locations), at 12° (8 locations), at 20° (4 locations), and at 30° eccentricity (8 locations). Stimulus radius is 3°.

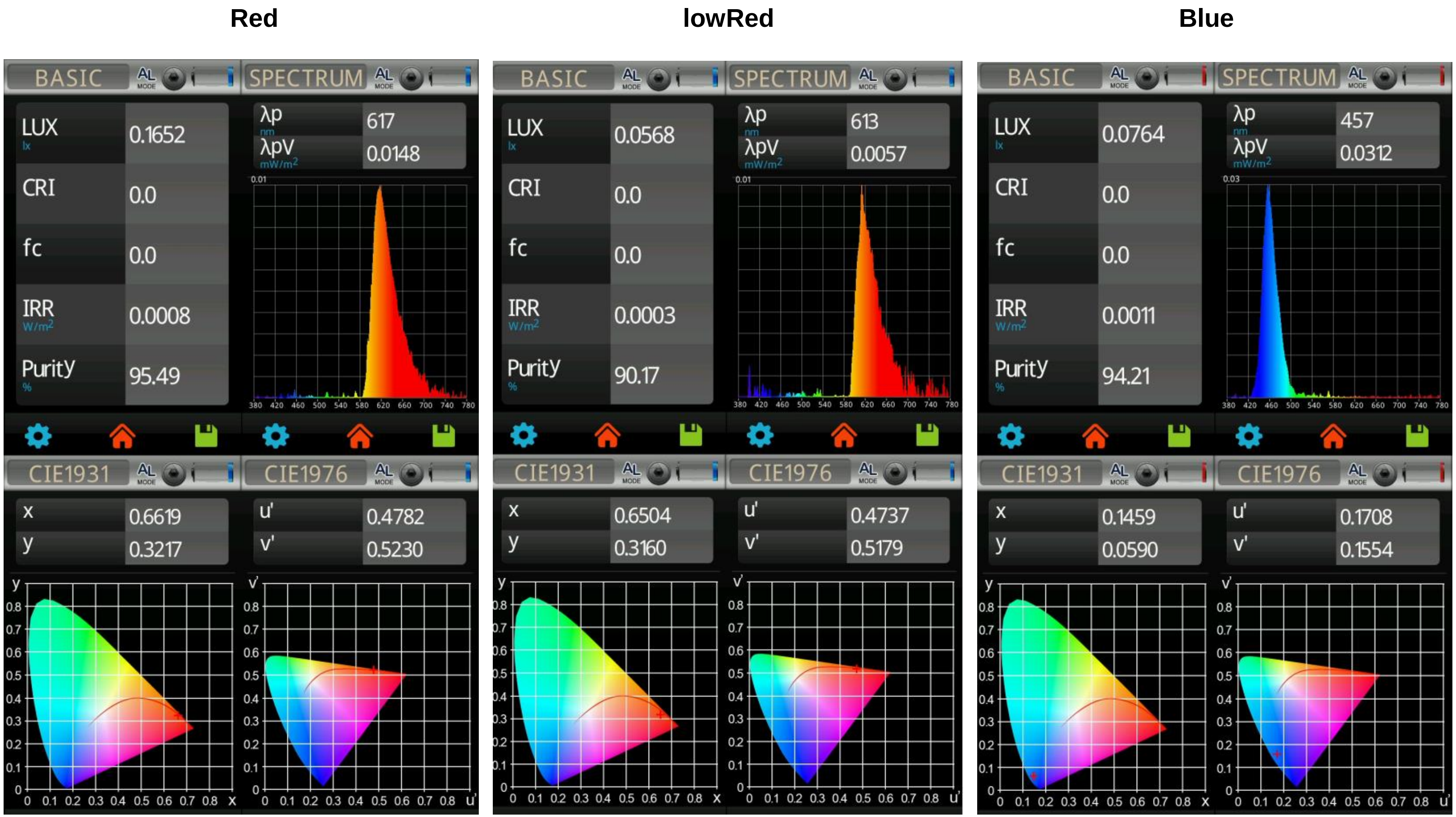

Supplement Figure 2 Energy and spectrum with CIE colour coordinates of the applied stimuli.
