# Supplementary material for: How lesions at different locations along the visual pathway influence pupillary reactions to chromatic stimuli
Source: Graefes Arch Clin Exp Ophthalmol. 2021 Dec 13;260(5):1675–85. doi: 10.1007/s00417-021-05513-5 (PMC9007757; doi:10.1007/s00417-021-05513-5)
Supplement: Supplementary file 2 — Supplementary file2 (PDF 61 KB) [file 417_2021_5513_MOESM2_ESM.pdf]

Supplement Table 1 Demographic and neuroophthalmological clinical data of all patients

	Age (y)	M/F	eye	Visual acuity (decimal)	Visual field defect	Funduscopy of optic nerve head (ON)	RNFL reduction in OCT	RAPD	diagnosis
Pre-01	67	M	L	0.5	Inferior scotoma	ON temporal pale	T, S, N	+	AION; OSAS
Pre-02	61	M	R	0.8	Inferior > superior scotoma	ON pale	TS >T, I	+	AION; Snoring (OSAS checkup not known)
Pre-03	69	M	R/L	0.8/0.5	R inferior > superior nasal scotoma;<br>L inferior scotoma	ON pale/ON pale	R S, N, TI;<br>L S, T	L +	AION R/L; OSAS
Pre-04	58	M	R	1.0	Inferior scotoma	ON nasal-superior pale	S, N	+	AION
Pre-05	75	M	L	0.8	Inferior scotoma	ON superior pale	S	+	AION; cardiovascular risks
Pre-06	59	F	R	0.25	Superior scotoma	ON temporal pale	T, TS, TI	-	AION R/L; OSAS
Pre-07	37	M	R	1.0	Superior scotoma	ON partially pale	TI, TS > T	n.k.	AION; OSAS
Pre-08	55	M	R	0.25	Inferior and superior scotoma	ON pale	Complete	-	AION R/L; (OSAS checkup n. k.)
Pre-09	69	M	R/L	1.0/0.16	Inferior scotoma L>R	R ON swollen;<br>L ON temporal pale	R swollen;<br>L T, TI, TS > N, NS	L+	AION R/L; OSAS
Pre-10	65	M	L	0.125	L inferior and superior scotoma	L ON pale	L complete	L+	AION R/L; OSAS
Chia-01	66	M	R/L	0.5/0.8<br>R amblyopic	Bitemporal hemianopia	R/L ON temporal pale	R N; L complete	-	pituitary macroadenoma;<br>resection 2014
Chia-02	78	M	R	0.63	Temporal hemianopia<br>(L amaurosis)	ON pale	complete	L+	pituitary macroadenoma;<br>resection 1990, 1997, 2010, 2012
Chia-03	37	F	R	1.6	Temporal hemianopia	ON temporal pale,	complete	L+	ruptured suprasellar dermoid cyst; resection 2012
Chia-04	31	F	L	1.25	Nasal incomplete hemianopia	ON temporal pale	T, TS, TI > N, NS	L+	bleeding cavernoma left (pre)chiasma;<br>resection 04/2019
Chia-05	43	M	L	0.25	Bitemporal in-complete hemianopia	ON temporal slightly pale	complete	L+	pituitary macroadenoma;<br>resection 08/2019
Chia-06	59	F	R/L	0.8/0.5	Bitemporal hemianopia;<br>R additionally defects nasal superior	R/L ON pale	R/L complete<br>(R N, NS > I > T, TS;<br>L T, N > S, I)	L+	pituitary macroadenoma;<br>resection 2010

Post-01	84	M	R/L	0.63/1.0	Right homonymous hemianopia	R/L ON vital	R/L normal	-	subacute ischemia left posterior cerebral artery
Post-02	54	M	R/L	1.6/1.25	Right homonymous hemianopia	R/L ON vital	R/L normal	-	left carotid dissection with left-hemispheric ischemia in the supply area of the middle and posterior cerebral artery with left internal carotid artery occlusion;<br>thrombectomy and stentimplantation in left internal carotid artery 2018
Post-03	68	F	R/L	0.8/1.0	Left homonymous hemianopia	R/L ON vital	R/L normal	-	Right occipital glioblastoma (WHO IV); resection 2017, radiation 2018, chemotherapy since 2018
Post-04	33	F	R/L	1.25/1.0	Left inferior homonymous quadranopia	R/L ON vital	R/L normal	-	cerebral right occipital ischemia due to CNS-vasculitis in Lupus erythematodes
Post-05	58	M	R/L	1.0/1.0	Left homonymous incomplete hemianopia	R/L ON vital	R/L normal	-	Occipital ischemia due to patent foramen ovale
Post-06	52	M	R/L	1.0/1.0	Left homonymous hemianopia	R/L ON vital	R/L T	-	Ischemia in the supply area of the right middle and posterior cerebral artery
Post-07	40	M	R/L	1.0/1.0	Right homonymous hemianopia	R/L ON vital	n.k.	-	Hemmorhagic apoplexia
Post-08 *	66	M	R/L	0.5/0.8	Left homonymous hemianopia	R/L ON temporal pale	R T, TI; S > NI;<br>L N, NS > T	-	Suprasellar and right parasellar craniopharyngeoma;<br>resection 2013
Post-09	47	M	R	1.0	Right and left upper quadrantanopia	ON vital	normal	n.k.	Bihemispheric ischemia due to vertebral artery dissection
Post-10 *	63	M	L	1.0	Left hemianopia	ON temporal slightly pale	N, NS, NI	L+	Clivus chordoma right extending to suprasellar region; subtotal resection and proton-radiation 2014
Post-11	54	M	R/L	1.0/1.0	Right homonymous incomplete hemianopia	R/L ON vital	R/L normal	-	cerebral ischemia
Post-12	80	M	R/L	0.8/0.8	Right homonymous hemianopia	R/L ON vital	n.k.	n.k.	Ischemia in the supply area of the left posterior cerebral artery
Post-13	48	F	Excluded due to missing CPC data → Examination terminated due to tumor-related circulatory problems						

M = male; F = Female; R = right eye; L = left eye; ON = optic nerve head; RNFL = retinal nerve fiber layer; OCT = optical coherence tomography; RAPD = relative afferent pupil defect; AION = anterior ischemic optic neuropathy; OSAS = obstructive sleep apnea syndrome; n.k. = not known

****Presumably tractus lesions***
